# Surgical Management of Renal Cell Carcinoma in Transplanted Kidneys—A Narrative Review

**DOI:** 10.3390/cancers17111864

**Published:** 2025-05-31

**Authors:** Oana Moldoveanu, Cătălin Baston, Adrian Traian Preda, Bogdan Sorohan, Robert Stoica, Cristian Mirvald, Ioanel Sinescu

**Affiliations:** 1 Department of Urology, Carol Davila University of Medicine and Pharmacy, 020021 Bucharest, Romania; oana.moldoveanu@drd.umfcd.ro (O.M.); cristian.mirvald@umfcd.ro (C.M.); ioanel.sinescu@umfcd.ro (I.S.); 2 Center of Surgical Urology and Kidney Transplantation, Fundeni Clinical Institute, 020021 Bucharest, Romania; a.t.preda@gmail.com (A.T.P.); bogdan.sorohan@umfcd.ro (B.S.); 3 Department of Nephrology, Carol Davila University of Medicine and Pharmacy, 020021 Bucharest, Romania

**Keywords:** renal cell carcinoma, allograft, kidney transplant, acquired cystic kidney disease, immune check point inhibitors, immunosuppression, nephron-sparing surgery, nephrometry scores, robotic surgery, ablative therapy

## Abstract

Renal cell carcinoma (RCC) is the most prevalent solid organ malignancy among kidney transplant recipients, demonstrating substantially higher incidence rates compared to those in the general population. Although RCC is most commonly diagnosed in native kidneys, its development in transplanted kidneys has an infrequent occurrence. The use of immunosuppressive therapies, pre-existing chronic kidney disease and the unique anatomical characteristics of transplanted kidneys represent considerable therapeutic challenges in managing RCC within this patient cohort. Open radical transplantectomy plays a crucial role in curative treatment for localized RCC, whereas nephron-sparing surgery (NSS), in selected cases, can provide similar oncologic benefits while preserving allograft function. Recently, laparoscopic and robotic surgical procedures have demonstrated favorable outcomes as viable alternatives to conventional open surgery. Furthermore, ablative therapies like radiofrequency ablation and cryoablation can be considered therapeutic alternatives for small renal masses, offering the benefit of preserving allograft function, especially in high-risk surgical candidates. Limited data exist regarding the management of metastatic RCC in transplant recipients. Surgery, withdrawal of immunosuppression and systemic adjuvant therapy could be considered. Management of RCC in transplanted kidneys requires a multidisciplinary approach considering patient-specific characteristics, tumor features and the developing landscape of both surgical and non-surgical options. Further research is needed to refine therapeutic strategies in order to achieve optimal oncological outcomes while preserving allograft function.

## Simple Summary

While the incidence of renal cell carcinoma (RCC) in kidney transplant recipients is higher than in the general population, surgical decision making, particularly in RCC in transplanted kidneys, is challenging due to immunosuppressive therapies, pre-existing chronic kidney disease and unique anatomical characteristics. This review aimed to evaluate risk factors and treatment options for RCC in transplanted kidneys using the most relevant studies from the PubMed database published between January 1999 and March 2025. Nephron-sparing surgery should be the treatment of choice for small allograft masses, providing favorable oncological outcomes while preserving kidney function. Laparoscopic and robotic partial nephrectomy techniques have demonstrated advantages such as reduced blood loss and shorter recovery time. Also, ablative therapies can be considered for small masses, especially in high-risk surgical candidates. Returning to dialysis after transplantectomy will impact the patient’s survival. Further research is needed to personalize oncological treatment strategies and improve both patient and graft survival.

## 1. Introduction

Kidney transplantation is the treatment of choice for patients with end-stage kidney disease (ESKD), improving overall survival (OS) and quality of life while being more cost-efficient than dialysis [1]. Cancer, with a 2- to 4-fold increased risk compared to that in the general population, is one of the most common causes of mortality among kidney transplant recipients [2,3]. Renal cell carcinoma (RCC) of native kidneys, and rarely allografts, is the most common urologic malignancy affecting kidney transplant recipients. There are no guidelines for RCC screening and treatment in this population [1,4]. However, the 2020 KDIGO guidelines (Clinical Practice Guideline on the Evaluation and Management of Candidates of Kidney Transplantation) recommend ultrasound screening only for high-risk candidates, including patients with dialysis history for more than 3 years, acquired cystic renal disease (ACKD) or analgesic nephropathy [5]. Currently, the main concern remains establishing optimal treatment strategies for RCC occurring in transplanted kidneys. Open nephron-sparing surgery (NSS) for RCC in kidney grafts has emerged as a viable approach for small tumors (<4 cm), demonstrating favorable oncological outcomes while preserving allograft function [2,6,7,8]. Although laparoscopic and robotic-assisted techniques have gained popularity due to their advantages of reduced blood loss and shorter recovery time, open surgery remains the optimal treatment in complex cases where tumor location and surgical objectives warrant extensive intraoperative visibility and control [9,10,11,12]. This article aims to review the complexities of RCC surgical management in transplanted kidneys, highlighting the importance of balancing oncological outcomes and kidney function preservation, while taking into consideration the unique characteristics related to transplantation and the evolving landscape of surgical techniques in this particular population.

## 2. Materials and Methods

This review aimed to evaluate the risk factors and treatment options for RCC in transplanted kidneys. The research was conducted using the PubMed database, focusing on studies published between 1 January 1999 and 24 March 2025. The search employed specific keywords to ensure relevance to the area of interest. The following keywords were utilized: “renal cell carcinoma”, “allograft”, “kidney transplant”, “acquired cystic renal disease (ACKD)”, “immune checkpoint inhibitors (ICI)”, “immunosuppression”, “nephron-sparing surgery (NSS)”, “nephrometry scores”, “laparoscopic surgery”, “positive surgical margins”, “robotic surgery” and “ablative therapy (AT)”. All types of studies published in English were included to provide a comprehensive overview of the subject matter. Articles were excluded if they were deemed irrelevant to the aims of the research or if they were not published in English. Among the 3457 articles initially found, 93 were ultimately selected. The review was informed by the most relevant oncology guidelines from the European Association of Urology (EAU), American Urology Association (AUA) and National Comprehensive Cancer Network (NCCN).

## 3. Epidemiology

RCC in kidney transplant recipients represents the most prevalent solid organ malignancy, comprising 6.8% of all malignancies diagnosed in this population [13,14]. The risk of developing RCC in kidney transplant recipients is estimated to be five to ten times higher than in the general population [15]. While RCC is predominantly found in native kidneys, representing up to 90% of cases, the occurrence of RCC in kidney grafts remains relatively rare, accounting for only 10% of cases [15,16,17].

A meta-analysis published in 2024 by Chang Xu et al. highlighted a significantly higher incidence of RCC following kidney transplantation compared to other solid organ transplants [18].

Further insights into the incidence of RCC in the kidney-transplanted population were provided by Chewcharat et al. (2019), whose meta-analysis reported an overall RCC incidence rate of 0.7% among kidney transplant recipients (95% Confidence Interval [CI]: 0.5–0.8%, I^2^ = 93%). Within this analysis, the incidence of RCC specifically arising in renal grafts was noted to be 0.2% [19]. Conversely, Barama et al. reported a higher incidence of RCC in kidney allografts (0.5%) [20].

Malignancies represent the third leading cause of death among kidney transplant recipients, with a RCC mortality rate accounting for 13.9% [19]. These concerning data highlight the need for vigilant surveillance and adapted management strategies to address cancer risk in these patients [3].

## 4. Risk Factors

The most important risk factors associated with RCC in kidney transplant recipients are shown in Table 1.

Immunosuppression has been incriminated as a significant risk factor for post-transplant malignancies. Azathioprine and Cyclosporine have been classified as human carcinogens by the International Agency for Research on Cancer (IARC), based on sufficient evidence linking their use to an increased risk of cancer in humans [2]. Limited data for the new immunosuppressive agents have not demonstrated their carcinogenic effects. Calcineurin inhibitors (CNI) are immunosuppressive agents used primarily to prevent organ transplant rejection and treat autoimmune diseases. Studies indicate that these drugs can activate RAS–RAF (rat sarcoma rapidly accelerated fibrosarcoma)-signaling pathway, which regulates cell proliferation and survival. This activation may also contribute to RCC tumorigenesis [2,21].

A large retrospective cohort study included over 1000 kidney transplant recipients, followed for 20 years, who received standard maintenance immunosuppression regimen revealed an increased malignancy risk among female patients with high cumulative doses of mycophenolate and young male patients with prolonged tacrolimus exposure. The cumulative incidence of primary malignant neoplasm demonstrated a progressive increase at 5, 10 and 20 years (4–5%, 10% and 25%, respectively) [2,3].

The Concept study, a systematic review and a meta-analysis encompassing 5876 kidney transplant recipients, demonstrated a 40% reduction in malignancy risk among patients converted to Sirolimus compared to those remaining on Cyclosporine. Additionally, kidney transplant recipients starting Sirolimus 3 months after transplantation had an increased mortality risk (43%) [22]. The TRANSFORM study evaluated low-doses of Everolimus combined with reduced CNI exposure in kidney transplant recipients and showed a lower incidence of malignancy in the Everolimus group compared to the control group receiving standard CNI dosing, while associating increased proteinuria [23]. Integrating these insights, clinicians must balance the risks and benefits of using CNI, particularly in patients at a higher malignancy risk, including patients with cancer history or predisposing conditions.

ESKD is an independent risk factor for RCC development [24,25]. According to the results coming from an international study, patients with ESKD have a 3.6 times higher risk of developing renal malignancies compared to non-ESKD patients [26,27]. Moreover, the cause of chronic kidney disease (CKD) could represent a risk factor for RCC after kidney transplantation. An elevated risk for RCC was found in kidney transplant recipients with vascular disease, glomerular disease and hypertensive nephrosclerosis. In contrast, patients with ESKD due to diabetes or polycystic kidney disease have a lower risk of developing RCC. These findings suggest that the underlying cause of ESKD might be related to the pathogenesis of RCC [26].

ACKD was reported as an important condition for RCC development [1]. The risk of ACKD and RCC decreases after kidney transplantation due to improved renal function. A cohort study that included 195 kidney transplant candidates and kidney transplant recipients evaluated the variation of cancer incidence in ESKD patients during periods of kidney function and non-function and demonstrated a greater risk for developing RCC on dialysis in both kidney transplant candidates and kidney transplant recipients with non-functional grafts. Therefore, kidney transplantation might have a protective role against developing RCC for patients with ACKD [6,28,29].

Kidney transplant recipients with a history of RCC have an increased risk of developing de novo malignancies or tumor recurrence, compared to those remaining on dialysis. Kidney transplant waiting time recommendations for patients with prior RCC are established according to KDIGO guidelines: For RCC < 4 cm, no waiting time is required; for RCC > 4 cm, up to 2 years; at least 2 years for tumors T3–T4; and patients with metastatic RCC or positive lymph nodes are not considered kidney transplant candidates, due to a high recurrence risk [30]. While a longer interval between malignancy treatment and kidney transplantation may improve oncological outcomes, patients remaining on dialysis have to face a higher risk of non-cancer related death [31,32]. Nephrectomy at the time of kidney transplantation for indolent RCC found in native kidneys showed no graft function impairment or survival impact, suggesting that a waiting time is not necessary [32,33].

Although risk factors for RCC in transplanted kidneys are not well established, a donor-derived etiology has been commonly proposed due to the higher incidence rates observed following deceased donor transplantation (74.2%) compared to living donor transplantation (25.8%) [34,35]. Despite their rarity, the complex etiopathology and challenging management of RCC in allografts have generated a considerable interest in the current literature. Penn retrospectively studied Cincinnati Transplant Tumor Registry and identified 45 cases of RCC in allografts among 9000 kidney transplant recipients [36]. Through a retrospective multicentric study also, Tillou et al. identified 79 cases among 41,806 patients [35].

Despite the elevated risk of RCC in allografts compared to that in the general population and the considerable risk of donor-transmitted malignancies, transplanting kidney grafts with small tumors does not increase cancer risk in selected recipients who otherwise would remain on the waiting list facing a higher mortality rate [37]. An Australian study, conducted by Nicol et al., reported only one recurrence of RCC at 9 years among 43 kidney transplant recipients who received kidney grafts either from deceased or living donors with small renal masses resected with negative margins on the back table before transplantation [38]. Musquera et al. reported no tumor recurrence at 32.34 months for 11 patients with transplanted kidney grafts from donors following excision of small renal mases incidentally found at the time of donor nephrectomy [39].

The time between kidney transplantation and RCC development in renal allografts varies from 9 to 312 months [35,40,41,42]. The 2-year cutoff to consider donor-transmitted RCC after kidney transplantation, as proposed by Penn [36], is debatable, since Park et al. confirmed the donor origin of the RCC in kidney grafts through DNA banding at 258 months after transplantation [40].

The donor-derived origin is important to evaluate when RCC in an allograft is detected. Localized RCC with a donor-derived pattern is considered stage 1, while RCC in allografts with host origin is considered metastatic disease. The paired recipient or the living donor should be screened for RCC when a donor-transmitted malignancy is suspected or confirmed [43,44]. Not all cases of RCC with recipient origin are metastatic, as Boix et al. reported a case of ccRCC with sarcomatoid changes in an allograft with recipient origin by DNA microsatellite analysis. No detectable tumor was found in the native kidneys by imaging, and immunohistochemical techniques excluded other potential tumor sites. In this case, “burned out” tumor origin was suspected [45].

**Table 1:** Risk factors for RCC in kidney transplant recipients.

Risk Factors Category	Details	Risk Level/Statistics
**Immunosuppression**	• Azathioprine and Cyclosporine [2] • Cumulative dose-dependent effect observed [2,3]	↑ Malignancy risk with cumulative doses [2,3]: **Mycophenolate:** ↑ risk in female patients **Tacrolimus:** Long-term exposure ↑ risk in young males
**Donor-Derived Malignancies**	Deceased donor transplants show higher incidence of RCC [34,35]	**74.2%** Deceased donors vs **25.8%** Living donors [34,35]
**ESKD**	Independent risk factor for RCC [24,25]	**3.6×** ↑ Risk of RCC vs. non-ESKD patients [1]
**Cause of ESKD**	**Higher Risk:** • Vascular disease • Glomerular disease • Hypertensive nephrosclerosis **Lower Risk:** • Diabetes • Polycystic kidney disease	Risk depending on underlying etiology [1]
**ACKD**	• 60% at 2–4 years of dialysis • 90% at >8 years of dialysis [46,47]	20% of patients with ACKD will develop RCC [1]
**Time Interval from Transplant**	• RCC occurrence ranges widely post-transplant [35,40,41,42]	**9–312 months** post-transplant [35,40,41,42] (2-year cutoff for donor transmission under debate—DNA tests to confirm donor-derived origin [36])

(ESKD—end stage kidney disease; RCC—renal cell carcinoma; ACKD—acquired cystic kidney disease). ↑—high.

## 5. Surgical Approach in RCC in Transplanted Kidneys

Currently, there are no established guidelines for treating RCC in kidney transplant recipients, reflecting the lack of consensus regarding malignancy management in this unique patient population. The absence of specific guidelines can be attributed to the complexities associated with the immunocompromised status of these patients, along with the intricate pathology of kidney transplantation and modified local anatomy. The interplay of various factors, including the evolving landscape of immunosuppressive therapies, advances in surgical techniques and the development of ablative therapies and adjuvant immunotherapies for RCC, has raised significant interest in identifying the most effective treatment strategies for these patients (Table 2).

Importantly, the staging system for RCC in native kidneys follows the same criteria as in the non-transplanted population as outlined by the American Joint Committee on Cancer (AJCC) [59]. However, for kidney graft RCC, the conventional staging system may not be applicable due to the unique anatomical and immunological considerations present in transplanted kidneys.

Tillou et al. elaborated a modified TNM staging system specifically adapted for kidney graft tumors although this system is not universally accepted by all authors [15,35,48]. The proposed staging system is presented in Table 3.

This modified staging system is important for determining the outcomes and management strategies for transplanted kidneys.

NSS has emerged as an effective alternative to nephrectomy for T1a tumors (tumors < 4 cm) in functional allografts, providing favorable oncological outcomes while preserving renal function [4,16,20,35,48]. According to data from the European Organisation for Research and Treatment of Cancer (EORTC), for RCC tumors < 5 cm there is no significant difference in cancer-specific survival (CSS) rates between partial nephrectomy and radical nephrectomy for non-kidney transplant patients [60].

Notably, radical nephrectomy for small renal tumors, while historically considered the standard treatment, has been associated with increased risk of cardiovascular mortality. This risk affects both non-kidney transplant recipients and kidney transplant recipients who return to dialysis after nephrectomy [52,53]. Consequently, preservation of kidney function through NSS becomes even more relevant in these patient populations.

The presence of positive surgical margins (PSMs) following NSS is considered a negative prognostic factor for oncological outcomes [61]. Studies indicate that the PSM rate after NSS for T1 staged tumors ranges from 2–8% [62]. Additionally, studies have shown that local bed recurrences occur in 16% of patients with PSMs compared to only 3% in those with negative margins, highlighting the importance of achieving clear margins during surgery. The RECORd-2 study identified hospital volume, specifically institutions performing more than 60 partial nephrectomies per year, as an independent predictor of PSMs [49]. This emphasizes the potential influence of surgical expertise and institutional experience on postoperative outcomes.

For kidney transplant recipients undergoing NSS for allograft RCC, available data showed that the rates of PSM and local recurrence are very low [34,50]. However, it is essential to note that much of this evidence derives from datasets featuring small patient cohorts or case reports.

Laparoscopic partial nephrectomy (LPN) has the advantage of lower blood loss compared to open partial nephrectomy (OPN), while ischemia time and operative time are longer [51]. Outcomes following 18 LPN cases on transplanted kidneys demonstrated maintained kidney function and no tumor recurrence during follow-up, with 100% survival at 78.6 months [12]. Similar oncological and functional results were recently reported by Trushkin et al. from a retrospective study on 28 kidney recipients with RCC who underwent LPN [9].

Robotic surgery has undergone important advances in the last decade, with remarkable results in renal surgery. ROBOCOP II, a randomized study with a 1:1 ratio, enrolled patients with localized RCC treated with OPN and robotic-assisted partial nephrectomy (RAPN). The robotic arm reported lower complication, reduced blood loss, decreased postoperative analgesic administration and shorter operative time and warm ischemia time [63]. Similar results were reported for RAPN and OPN performed for allograft RCC in a retrospective study which included 11 patients (OPN—5 patients, RAPN—6 patients). The mean nephrometry score was 9 in the OPN arm and 5 in the RAPN arm. Negative margins were obtained in both arms. The mean follow-up period differed between the two arms (124.2 months for OPN vs. 18.8 months for RAPN). One recurrence was reported in the RAPN arm at 35.3 months. In the OPN arm, one case was staged pT3a with no recurrence during follow-up. The variation of eGFR in both arms was similar. Lower intraoperative blood loss and a shorter hospital stay were observed in the RAPN arm. One case of ureteral injury was reported in each arm, and one patient in the OPN arm experienced graft loss at 4.8 months [10]. Kaouk et al. reported similar oncologic and functional results for one case of a cT1b tumor treated by RAPN [11]. Despite these findings coming from small cohort studies, RAPN may be an optimal treatment for cT1a RCC in kidney grafts [10].

Off-clamp vs. on-clamp partial nephrectomy, according to the CLOCK trial, have comparable results on kidney function [64]. Clamping and partial nephrectomy for RCC in kidney grafts, however, is a more complex surgery. Significant challenges are encountered in dissecting the internal or external artery, isolating the graft artery and positioning the bulldog clamp. Ureteral dissection may also be difficult after postoperative adherences and necessitates high precision to avoid ureteral injuries [10,11,34,48,65].

The surgical complexity is also influenced by the tumor position. Moreover, no nephrometry scores for kidney grafts to evaluate the surgical outcomes are available; most of the authors appreciate tumor complexity with native kidney nephrometry scores, despite the anatomical changes in the allograft and iliac fossa [11].

Nephrectomy is indicated for RCC occurring in native kidneys, non-functional grafts, locally advanced or metastatic RCC and multicentric papillary RCC to achieve good oncologic outcome. For complex localized tumors, transplantectomy is a feasible option to reduce PSM rates and decrease the risks of recurrence, urinary fistula and blood loss. Individual patient evaluation must balance the risk of returning to dialysis and oncologic prognosis, taking into consideration that the presence of malignancy precludes future kidney transplantation [66,67,68,69,70,71].

An important aspect for oncological outcome that has not been evaluated yet, is the role and pattern of lymph node dissection (LND) for RCC occurring in kidney grafts, taking into consideration that LND for clinically positive lymph nodes for RCC in healthy native kidneys demonstrated important improvement in OS in a specific subcategory of patients (cT3-T4NxM0) [72]. Despite the fact that LND is still controversial, it plays an important role in staging, follow-up and adjuvant therapy [73,74].

Over the past two decades, systemic therapy for metastatic renal cancer has undergone substantial changes. The approach has transitioned from first-generation immunotherapies like interferon and interleukin-2 to targeted treatments that include anti-VEGF antibodies and immune checkpoint inhibitors (ICIs). Recent clinical studies demonstrate that regimens incorporating ICIs provide superior outcomes compared to the earlier standard treatment with sunitinib. Notably, the likelihood of achieving a complete response with sunitinib is around 1%, while therapy with ICIs offers an increase of over 10% in the chance for a complete response [57,58]. For kidney transplant recipients, most of the data on ICI administration comes from systematic reports, case series and case reports and raises concerns due to the high risk of graft rejection (41–48%).

The rejection rate is higher among kidney transplant recipients, compared to liver, heart and lung transplant patients. Eight years post-transplantation, the use of 2 immunosuppressive agents or mTOR inhibitor-based therapy for deceased donor recipients were associated with lower rejection rates after ICI treatment in kidney transplant recipients [75,76]. In a systematic review on ICIs in solid organ transplanted patients, partial or complete tumor response was achieved in approximately one-third of patients and was not correlated with immune-related adverse events, time from transplantation, specific immunosuppressant medications or instances of rejection. According to the same study, the use of at least one immunosuppressant drug other than steroids was linked to reduced rejection rates, but also exhibited a trend toward lower progression-free survival. The limitations of studies assessing graft rejection and tumor response in kidney transplanted patients receiving ICI are represented by small sample size cohorts and inclusion of most patients with types of cancer other than RCC [76,77]. The use of tyrosine kinase inhibitors (TKIs) for metastatic RCC in kidney transplant recipients is also limited to few case reports [78,79]. No guidelines or clear recommendations exist for reducing, discontinuing or modifying immunosuppression regimens. For metastatic RCC in transplanted kidneys, cytoreductive nephrectomy, immunosuppression withdrawal and administration of systemic adjuvant therapy should be considered the milestone of treatment [80].

## 6. Pathology Findings

Clear cell renal cell carcinoma (ccRCC) is the most commonly identified subtype of RCC in transplanted kidneys, followed by papillary renal cell carcinoma (pRCC) [1]. The incidence rates of these two cancer types differ significantly from those observed in the general population. Notably, the incidence of pRCC in transplanted kidneys is markedly higher compared to that in the general population (42.1% vs. 1–15%), while ccRCC had a lower incidence in transplanted kidneys (45.7% vs. 75–80%). While the increased risk of pRCC in the native kidneys of patients with ACKD due to ESRD is well documented, the elevated risk of pRCC in renal allografts has yet to be established [34,81]. Chromophobe and translocation RCC in the allograft are rarely reported [7,8]. According to the grading system, most of the tumors in transplanted kidneys tend to be low grade, Fuhrman 1 and 2 in most of the cases [2,34,35,48].

## 7. Alternatives to Surgery

Biopsy is required prior to ablation to avoid treatment of benign renal masses, considering that up to 45% of patient who received ablative therapies were treated for benign or non-diagnostic tumors [82,83]. The 5-year disease survival depends on the subtype of RCC (90% for ccRCC, 100% for pRCC), according to one study, for 2.5 cm T1a tumors treated with radiofrequency ablation [84]. Liu et al. found that tumor size greater than 4 cm is a risk factor for recurrence after percutaneous radiofrequency ablation (PRFA). The 10-year OS was lower (50%) in patients with ccRCC and tumors > 4 cm treated with PRFA compared to patients treated with partial nephrectomy; in contrast, patients with non-ccRCC and tumors > 4 cm treated with PRFA had a 10-year OS comparable to that of partial nephrectomy (100%) [85]. Cryoablation shows promising oncologic results in a retrospective study published by Breen et al. on 484 patients with T1a tumors, showing 96% efficacy after primary treatment and 98% after secondary cryoablation treatment [86]. In contrast, local tumor control for cT1b tumors is 60.3% at 3 years [87]. Endophytic RCC, besides tumor dimension, is a predictor of recurrence (11×) [88]. For cT1a tumors, ablative therapies have similar recurrence-free survival rates compared to partial nephrectomy. However, the metastasis-free survival rate and OS are better for partial nephrectomy. The limitations of ablative therapies include the inability to obtain a definitive histology, impossibility to evaluate oncologic margins and challenges in the follow-up strategy to evaluate recurrence and distant metastasis. These results must be interpreted with caution due to selection bias and retrospective study design [65].

In kidney transplant recipients who develop RCC in allografts, ablative therapies have been utilized more frequently in recent years to prevent the potential complications of partial nephrectomy and avoid dialysis after transplantectomy [89]. In a large systematic review conducted by Favi et al., pRCC was the most frequently treated tumor using ablation techniques (AT), with most lesions measuring less than 4 cm and classified as T1a N0 M0. The majority of these tumors were endophytic, while two tumors exceeded 4 cm (T1b N0 M0), one of which required partial nephrectomy after unsuccessful AT. Most of the tumors were graded Fuhrman 1 or 2. The postoperative complication rate was low, with only two cases requiring additional surgical intervention. Allograft function was well-preserved in almost all patients. AT showed effective and safe outcomes, with only three primary treatment failures and a single local recurrence [42]. The optimal approach for confirming complete tumor ablation and monitoring for local recurrences remains under discussion, as follow-up protocols differ considerably among centers [42,89,90].

Active surveillance could be an option in selected cases, due to the high prevalence of pRCC, which has a lower growth rate (0.02 cm/year vs. 0.25 cm/year for the ccRCC) and a lower risk of progression to metastatic disease [91]. For kidney transplant recipients with graft RCC, intensive follow-up evaluations must be conducted, due to a higher progression rate (0.5 to 7.5 cm/year) [54,55,56]. Notably, no active surveillance strategy has been established to manage RCC in transplanted kidneys.

## 8. Conclusions

The surgical management of RCC in transplanted kidneys poses unique challenges due to the interplay of oncological and transplant-related factors. NSS has been established as an effective and preferred approach for T1a tumors < 4 cm in functional grafts, preserving allograft function while providing good oncological outcomes. Recently, robotic and laparoscopic approaches reported lower complication rates and similar oncologic outcomes. Although AT might be considered alternative treatment options, the long-term efficacy and risk of recurrence associated with these methods need to be evaluated in randomized control trials. Further research is needed to personalize oncological treatment strategies and improve both patient and graft survival.

## Figures and Tables

**Table 2:** Treatment-adapted strategies for transplanted kidneys with RCC.

Treatment Option	Description	Advantages	Limitations	Outcomes
**Open Nephron-Sparing Surgery (NSS)**	Surgical procedure preserving kidney function for T1a tumors (<4 cm). [4,16,20,35,48]	↑ Oncological outcomes ↑ Renal function [4,16,20,35,48]	↑ Surgical complexity [49]	**Local recurrence: 3.6–6%** [34,50]
**LPN, RAPN,** **NSS**	Surgical alternatives.	↓ Blood loss ↑ Functional outcomes [51] ↓ Complications (RAPN) [9]	↑ Ischemia time (LPN) [51] ↑ Operative time [51] ↑ PSM [51]	Comparable oncological outcomes among LPN, OPN, RAPN [9]
**Ablation Techniques**	Minimally invasive techniques: **• Radiofrequency ablation** **• Cryoablation**	Preserves renal function Less invasive procedure Repeatable if necessary [52,53]	No definitive histology ↑ Treatment failures + Smaller lesions [52,53] Close follow-up [52,53]	↑ **Efficacy for T1a tumors** [52,53] ↓ Local control rates for T1b [52,53]
**Active Surveillance**	Monitoring small, low-growth tumors without immediate intervention. [52,53]	pRCC—low risk of progression [52,53]	Requires intensive follow-up ↑ Disease progression [54,55,56]	No data available
**Radical Nephrectomy**	Standard treatment for larger tumors (>4 cm) or complex cases where NSS is not feasible. [52,53]	Complete tumor removal	↑ Risk of cardiovascular mortality [52,53] Loss of kidney function Return to dialysis	↑ Oncological outcomes [52,53] ↑ **Complication risk** [52,53]
**Systemic Therapy (Immunotherapy,** **TKIs)**	Treatment for metastatic RCC including ICIs and TKIs. [57,58]	Especially effective with ICIs [57,58]	↑ Risk of graft rejection Limited data on RCC outcomes in transplant patients [57,58]	↑ OS [57,58] Promising results from limited data [57,58]

(NSS—nephron sparing surgery; LPN—laparoscopic partial nephrectomy; RAPN—robotic-assisted partial nephrectomy; OPN—open partial nephrectomy; PSM—positive surgical margins; TKI—tyrosine kinase inhibitors; ICI—immune checkpoint inhibitors; RCC—renal cell carcinoma; pRCC—papillary RCC). ↑—high; ↓—low; +—positive oncological results.

**Table 3:** Staging system of RCC in transplanted kidney according to Tillou et al. [35,48].

Allograft RCC Staging System	
**T1**—tumors equal to or less than 7 cm confined to the kidney	**T1a** tumors: ≤4 cm **T1b** tumors: >4 cm but <7 cm
**T2**—tumors that exceed 7 cm while still being confined to the kidney	
**T3**—tumors are defined by extension into major veins or invasion of renal sinus fat or peritoneum	**T3a:** Tumors that invade renal sinus fat or peritoneum **T3b:** Tumors invading the external iliac vein or common iliac vein **T3c:** Tumors that invade the inferior vena cava
**T4**—tumors that invade surrounding perinephric organs, such as the psoas muscle, walls of the iliac vessels, bladder, small intestine and colon	
